# Employee Management in Dairy Farms Associated with Bulk Tank Somatic Cell Count and New Mastitis Infection Risk

**DOI:** 10.3390/vetsci11120646

**Published:** 2024-12-13

**Authors:** Michael Farre, Erik Rattenborg, Henk Hogeveen, Volker Krömker, Carsten Thure Kirkeby

**Affiliations:** 1 SEGES Innovation, Agro Food Park 15, 8200 Aarhus, Denmark; 2 Business Economics Group, Department of Social Sciences, Wageningen University and Research, 6706 KN Wageningen, The Netherlands; 3 Department of Veterinary and Animal Sciences, Section for Production, Nutrition and Health, University of Copenhagen, 1870 Frederiksberg C, Denmark

**Keywords:** new infection risk, employee management, training employees, SOPs

## Abstract

For decades, bovine mastitis and milk quality have been a focus area for research, agricultural extension, and dairy processors worldwide, yet employee management as a factor in udder health management has received limited attention. This is mainly because the focus has previously been on more classical areas covered by the National Mastitis Council Mastitis Control Program (NMC 10-point plan) in English-speaking countries. Therefore, we wanted more background information on employee management on dairy farms, to identify the human factor of udder health management. The method of investigating employee management and the impact of employee management on udder health was conducting a study of 88 Danish dairy farms with hired employees and parlor or rotary milking systems. An interview-based questionnaire on individual dairy farmers’ human resource management was developed based on the current literature and multiple discussions among the authors. The results we found through analyzing associations between the dependent variable BTSCC and employee management, using a regression model, was that providing a generic SOP was associated with a 21,600 cells/mL increase in BTSCC, with estimates in the range (507; 42,674 cells/mL). We also analyzed, applying a Poisson model, that there was a 0.16% reduction in new infection risk if the training was based on a herd-specific SOP and educated employees. In contrast, we identified a 0.15% increase in new infection risk in herds where SOPs were available but not incorporated, both modest but significant results. In conclusion, farms with educated employees and trained by an SOP achieve the lowest new infection risk, but education has no impact on BTSCC.

## Simple Summary

This study examines the impact of employee-related factors on udder health in dairy cows. Specifically, it investigates how variables such as educational level, the nature and scope of training provided, the methodologies utilized in that training, and the adherence to Standard Operating Procedures (SOPs) within a dairy herd influence Bulk Tank Somatic Cell Count (BTSCC) and new infection risk. We anticipated that employees with formal education and comprehensive training in SOP implementation would be associated with lower BTSCC and new infection risk than their counterparts lacking formal agricultural education. Our findings indicate that the presence of an SOP correlated with an increase in BTSCC by 21,600 cells/mL compared to herds lacking a milking SOP. Furthermore, we observed a reduction of 0.02% in new infection risk when employing educated staff, which decreased to 0.16% when an SOP was incorporated into the training regimen. In contrast, unskilled employees were linked to a 0.02% increase in new infection risk. Notably, the availability of an SOP in the herd that was not incorporated within the training was associated with a 0.15% increase in infection risk. These results underscore the importance of combining foundational knowledge with implementing SOPs in training programs to optimize milking protocols.

## 1. Introduction

Even after decades of interventions and improvement, the global dairy sector still faces substantial losses due to bovine mastitis [1,2]. The latest estimate, which highlights the potential for improvement, has estimated global losses of approximately USD 13B for clinical mastitis and USD 9B for subclinical mastitis [3]. Furthermore, a dairy cow with subclinical or clinical mastitis needlessly compromises the environment with increased emissions [4]. This additional negative impact is of growing importance due to the significant focus on milk production in the global climate debate [5]. Therefore, bovine mastitis still presents many challenges that must be addressed if the dairy sector is to meet the demand for quality milk, reduce the consumption of antimicrobials, and work to improve climate sustainability. The reduction is important because 80% of antimicrobials are used in dairy animals, according to Touza-Otero et al., 2024 [6]; therefore, targeted treatment of clinical mastitis has increasing importance for the consumption of antimicrobials.

Research has, to date, mainly focused on the pathogen as a vital factor for mastitis [7,8,9]. Pathogens that cause mastitis can be contagious or environmental by nature but also have similar intramammary infection (IMI) dynamics. The common characteristic, however, is that they lead to a loss in milk production [10]. In addition, farmers have shifted from focusing on individual animals to a more herd-oriented approach [11]. For years, interventions to improve udder health have, in many English-speaking countries, been driven by the NMC 10-point plan [12]. The NMC 10-point plan was developed by the National Mastitis Council, with the intention of highlighting the most important interventions to improve udder health, but the plan focuses predominantly on contagious mastitis and less on, for example, motivation, implementation, and the employee factor. Therefore, past efforts have mainly regarded factors like teat disinfection, culling of chronically infected cows, and blanket dry cow therapy as essential for mastitis management [12]. There has been extensive research on the risk factors related to mastitis and udder health during various stages of lactation, but little attention has been paid to employee management [13,14,15]. Long working shifts in the milking parlor can lead to low job satisfaction and increased risk of employee turnover, which can negatively impact the quality of work [16]. While dairy farmers have been aware of the growing emphasis on human resource management for years, there is still a lack of comprehensive information on this issue from research and extension services [17]. Regular expansion adds to the challenges caused by increasing herd sizes, where owners regard the growing labor force as the most demanding aspect of the expansion process [18]. Alongside growing herd sizes, the dependence on hired employees is mainly based on migrant employees [19].

A fundamental part of human resource management when improving the profitability of dairy farms is communicating verbally and in writing and providing “hands-on” training within the core areas of dairy farming, such as milk quality [20]. A generic SOP is often provided by many of the suppliers to a dairy farm; they are developed as “one size fits all” and therefore should be avoided [21]. On the contrary, a herd-specific SOP, developed and designed for a specific herd, based on evidence-based principles is the appropriate approach to implementing SOPs. A focus on employee training and implementing herd-specific SOPs as means of improving performance in udder health was also the finding of other researchers [22]. Therefore, employees with good relationships with their supervisors are essential for job satisfaction and the possibility of recruiting new colleagues [23]. This relationship and job satisfaction are important because the profitability and productivity of the dairy farm is indirectly linked to the performance of udder health and clinical mastitis [24]. Also, regular training and supervision is important to ensure consistency in milking work in the parlor [25].

Therefore, dairy farmers are facing a growing dilemma. They are spending an increasing amount of time managing employees, and the employees are managing the cows. Thus, the dairy farmer must be skilled in both human and cow management. Motivated by these challenges faced by dairy farmers, and to investigate the impact of employee management on udder health, we conducted this study on education levels and how training is provided in Danish dairy herds to explore the association between the explanatory variables, educational background, training sessions, training method, and work instruction, and the dependent variables of BTSCC and new infection risk at herd level. When evaluating the current management practices in the herd, bulk milk samples are fast and inexpensive snapshots of the present situation [26]. Therefore, as suggested by Schukken et al. (2003) [27], we applied the standard proxy for udder health at a dairy farm, BTSCC, to identify an association with employee management. In addition, we also applied the new infection risk from Dairy Herd Improvement (DHI) to indicate udder health on a farm level, as suggested by Dohoo and Leslie (1991) [28]. Our focus was on herds with employees milking in a parlor or rotary system, where the employee training and management had a direct impact on milk quality. Therefore, we conducted a qualitative questionnaire study and added retrospective data from the national cattle database with the farmer as study unit, where we hypothesize that hiring educated employees, defined as employees with an agricultural college background, and providing training using herd-specific SOPs would be associated with a reduction in BTSCC and new infection risk. We therefore expected to find a negative association both between BTSCC and new infection risk and dairy farms where the farm employees were educated personnel trained using an SOP. Our objectives were to evaluate the association between employee management in dairy farms and (1) BTSCC and (2) new infection risk.

## 2. Materials and Methods

The study populations were dairy farms, and the target populations were Danish dairy farms. As the first step, we defined several essential enrolment criteria to target the predominant herd type in Denmark: milking in a parlor/rotary, >90% pure-breed Holstein cows, no organic dairy herds, milk recording (DHI recording), herd size > 100 cows, and postal code > 6000. We then decided to focus the fieldwork on the western part of the country, where most dairy herds are located. These criteria were selected because we wanted herds with employees milking, therefore the choice of parlor/rotary. Holstein, because it is the dominant breed in the country, and conventional herds, because they are non-grazing confinement herds. The choice of herd size was the minimum where we expected they would have employees, and the postal code was, for convenience, in the Western part of the country.

In the current study, the calculated minimum sample size was 218 dairy farms, but we enrolled 88 dairy farms of eligible dairy herds due to limitations in resources and time. This sample was convenient in terms of the cost and logistics associated with the 498 eligible dairy herds. The first author contacted all the enrolled dairy farmers and conducted the interviews to ensure consistency in the data collection. Each dairy farmer was initially contacted by phone, and a maximum of two attempts were made to get in touch before moving on to the next potential participant. They were encouraged to participate in the project, which included a farm visit, at which the first author interviewed the person responsible for labor management. The dairy farmer received information about the purpose and aim of the project, where we stressed our particular interest in employee management, whereafter two declined to participate due to lack of time. All the dairy farmers signed a declaration to consent to the interview, to ensure access to DHI data for research, and to approve the use of anonymized interview data in this study. The herd veterinarian was also informed about the aim and objectives of the project, even though they were not directly involved in this study.

### 2.1. Questionnaire

A questionnaire on individual dairy farmers’ human resource management was developed based on multiple discussions among the authors. The questions were centered on four categories: education, employee training, training method, and work instruction. We wanted to acquire more information on the actual levels of educational background because the employees in dairy farming often have low-level entry jobs. Still, we believe that many dairy farms’ size, and complexity require employees with technical and theoretical knowledge in animal welfare and management to achieve good udder health results. Next, we wanted to document the approach to training, both in terms of structure and method, because the success of the implementation very much aligns with the method applied. Last, we wanted to evaluate if the management procedures in dairy herds were performed based on an organized method like an SOP.

The questionnaire was tested in a pilot study setup with colleagues and dairy farmers to ensure understanding and relevance of the questions. Based on the pilot study, the answer option “other” was added to the questionnaire answer options in Table 1.

To ensure consistency in data collection, we used an Excel^®^ spreadsheet with predefined dropdown menus to record the closed-end answers. This was done to reduce errors in typing and facilitate subsequent data management. We chose four categories of aggregated answer options to reveal the approach to managing employees at the dairy farm. We first identified the educational background based on agricultural collage training, because we expected that employees with a documented training background had been trained and understood basic udder health management related to milking in a parlor/rotary. Also, previous experience/skills are an issue, but assessment of the quality of this is highly subjective. Therefore, we focused exclusively on the training they received in the herd, because milking procedure and routines must be herd-specific and not based on generic experience. The next three categories describe how procedures were implemented by dairy farm management through the employees, as illustrated in Table 1.

We asked about the distribution of employees attending agricultural college and employees with no formal agricultural education, if they had farm-specific training sessions for the employees, if they had training sessions of one of the four methods mentioned in Table 1, and, finally, if they had SOPs for describing the task the employees had to perform.

### 2.2. Statistical Analyses

The dependent variable for the first analysis was the last 12-month average geometric BTSCC as an indicator for udder health over time at herd level, as described by Schukken et al. (2003) [24]. The dependent variable for the second analysis was the average new infection risk in the herd during lactation over the previous 12-months, defined as the mean of the proportion of cows changing from an Somatic Cell Count (SCC) at cow level below 200,000 cells/mL to one above 200,000 cells/mL between two DHI tests, to analyze the dynamics in the herd, as suggested by Valde et al. (2005) [29]. We also included herd explanatory variables (Table 2) from the Danish National Cattle Database [30] to strengthen this study further. These data were accessed directly in the database with the permission of the dairy farmer and included the herd average energy-corrected 305-day milk yield (Herd average ECM 305), herd average culling rate over 12 months (Culling rate, cows removed from the herd), and average herd size of dairy cows over the previous 12 months (Herd size). Previous studies found these variables to be associated with mastitis and thus the SCC and new infection risk at cow level [31,32]. The management variables included in the analysis are educational level, training session, training method, and work instruction, as in Table 2. The levels and questions included are the explanatory variables we expect to have an impact on new infection risk subsequent to the BTSCC.

Data management and subsequent analyses were conducted in R (Version 4.2.0, R Core Team 2022, https://www.R-project.org/, (accessed on 15 June 2022)), and explanatory variables with *p* ≤ 0.05 were considered statistically significant. Linear regression analyses were performed on a normally distributed dataset in a backward elimination process to identify the association between management procedures and BTSCC as the continuous dependent variable. Next, the data for new infection risk were exponentiated and plotted to evaluate normal distribution residuals and homoscedasticity. The data for new infection risk were regarded as count data; therefore, Poisson regression analyses for modelling rates were performed and added as an offset, to identify the association between management procedures and new infection risk as the dependent variable. For each herd, the new infection risk was calculated based on retrospective data from the last 11 DHI tests during the last 12 months before the herd visit. The explanatory variable educational background had a binary outcome, and, if the dairy herd had more employees, the distribution between them was considered in the model. We calculated a full-time employee as having a 37 h a week as their expected work input. Six separate models were then set up to explore the data further, three with BTSCC as the dependent variable and three with new infection risk as the dependent variable, as illustrated in Table 2.

First, we explored the herd and management explanatory variables separately (Table 2). This was performed using a linear regression model to analyze the association with BTSCC, where our variables were independent, and the linearity between the dependent and independent variable were linear. Next, we developed a Poisson regression model to analyze the association with the new infection risk, because our response variable of new infection risk represents the number of events occurring within our retrospective dataset 12 months prior to the visit, and the variable is independent. Then, we built two models with only the herd-related explanatory variables of herd average ECM 305, culling rate, and herd size, with either BTSCC or new infection risk as dependent variables. Then, we built two models with the employee management explanatory variables: one with BTSCC as the dependent variable and the other with new infection risk as the dependent variable. Finally, we built two combined models with herd and management as explanatory variables to verify whether the results of the management models were robust to the inclusion of herd-related explanatory variables, as in Equations (1) and (2).

BTSCC ~ Herd average ECM 305 + Culling rate + Herd size + Educational background + Training session + Training method + Work instruction (1)

New infection risk ~ Herd average ECM 305 + Culling rate + Herd size + Educational background + Training session + Training method + Work instruction (2)

A backward elimination procedure identified the explanatory variables that significantly affected the dependent variables, where the explanatory variables with a statistically significant association were retained in the model.

## 3. Results

We enrolled 88 herds in this study, equal to 18% of the study population, with herd sizes ranging from 105 to 1.291 cows and an average of 326 milking cows, while the number of full-time employees assigned to the milking cows ranged from one to twenty-one. Both BTSCC and new infection risk were identified as normally distributed. The BTSCC 12-month geometric mean, median, and quartiles, as well as the herd’s 12-month average new infection risk, mean, median, and quartiles, were calculated as illustrated in Table 3.

Table 4 shows the results of the linear regression modeling, including management and herd variables, and illustrates the full and final model after backward selection for the dependent variable BTSCC. The management variables can be seen in the Supplementary Material Figure S1). In the model, we found two explanatory variables which turned out to be statistically significant according to the decided level *p* ≤ 0.05. In the final model, the significant explanatory variable was the availability of an SOP in the herd, with an intercept at 196,614 cells/mL and an increase in BTSCC of 21,590 cells/mL. The second significant explanatory variable that impacted the BTSCC was herd average ECM 305. Every time that the herd average ECM 305 increased by 1000 kg, the BTSCC was reduced by 15,000 cells/mL.

Table 5 illustrates the result of the modeling for new infection risk as a dependent variable, with a threshold of 200,000 cells/mL for calculating the infection status, as suggested by Dohoo and Leslie (1991) [25].

The new infection risk was associated with several of the explanatory variables. The mean new infection risk, as seen in Table 3, was 11%, and the new infection risk in herds where SOPs were available was 0.15% higher, in contrast to a 0.16% reduction if the training was done with a herd-specific SOP.

## 4. Discussion

Studies like this are labor-intensive but very useful for understanding and investigating the impact of employee management on udder health status. Only very few studies have focused on employee management as a risk factor for udder health as described by BTSCC and new infection risk. With the resources available to us, we enrolled 88 dairy farms in our study, equal to 18% of the study population, which was less than our calculated sample size of 218 dairy farms. The number of enrolled farms compromised the power of this study, but the significant results we obtained can anyway be regarded as very robust with less than half of the calculated sample size.

The enrolled herds can cause concerns in terms of the sociodemographic characteristics and factors associated with BTSCC; based on the initial selection criteria for participating, we had 498 eligible herds; that sample was randomized before contact and is within the average herd size in the Western part of country. We are aware of some of the limitations and biases of this study, based on the enrollment criteria we chose. If we tried to compare udder health with a similar group, it would be dairy farms with robots, but, here, the employee’s direct impact on udder health is limited because they are not milking the cows. Also, we selected the Western part of the country, where the largest farms are situated, and farm size could have an impact on the results as well; larger farms will have better options to implement procedures and systems than smaller farms. Similarly, organic farms will be biased by limitations on treatments, and, in this country, they have several limitations on excipients; therefore, comparing udder health to conventional dairy farming would be problematic. The self-reporting data we collected can also be biased by several factors which impact on the results in the conclusion that employing educated employees gives better results in terms of new infection risk. The dairy farmer could report a higher number of employees with an agricultural college education because social norms usually prefer educated people, but also very often, in reality, they need to employe migrant employees. Also, there could have been recall biases where they cannot remember all employees, considering we had dairy farms with up to 21 employees. Lastly, we could have improved the data with timed questions and confirmations to ensure people were answering the questions accurately.

We had a response rate of 100% among the dairy farms that participated in this study, which is high for such studies, but can be explained by the on-farm visit—with only two declining.

We assessed the association between employee management in terms of educational background, training sessions, training method, and work instruction with the two dependent variables of BTSCC and new infection risk at herd level. We have not found similar studies that directly linked employees with BTSCC and new infection risk, but previous studies have shown that employee turnover is a risk factor directly related to the success of a dairy operation [20,23,33]. Thus, a study from the US with a focus on training milking employees resulted in a reduction in inadequate prep-lag time, improved teat cover of teat disinfection post-milking, and, lastly, increased detection of clinical mastitis (Rodriguez, Zelmar 2024). These variables indirectly impact the BTSCC and new infection risk, and, presumably, result in a reduction in new infection risk due to improved milking and post-milking teat disinfection [34]. As stated by Billikopf. 2003 [35], “Performance is often enhanced when employees believe they are contributing to a valuable product and are part of an effective team”. Therefore, a focus on the training and motivation of the milking team is essential for the success of the dairy.

The BTSCC model showed, surprisingly, that having an SOP available was generally associated with an increase in BTSCC of 21.600 cells/mL when it was not incorporated through herd-specific training. The other significant explanatory variable in the BTSCC model was the herd average ECM 305, with a decrease of 15,000 cells/mL for each 1000 kg increase in herd average ECM 305, which is a statistically significant but overall minimal contribution to the BTSCC dynamics. The rise in herd average ECM 305 is related to the management level in the herd, and it can therefore be assumed that the increase in milk yield is catalyzed by the general management level of the dairy farm. Thus, we expected to identify a significant effect on education level and BTSCC, but our interpretation of the current result is that BTSCC can be controlled by separating milk, which does not require a lot of education to implement; therefore, the education level does not result in improved BTSCC.

In the new infection risk model, we found a positive and significant association between new infection risk and the explanatory variables of skilled employees, with a 0.02% reduction, and using and training with an SOP, with a 0.16% reduction in new infection risk. In contrast, there was a negative and significant association between new infection risk and unskilled employees, with 0.02% increase in new infection risk; being trained by other employees, with a 0.05% increase in new infection risk; being trained by farm owners, with a 0.11% increase in new infection risk; and having an SOP provided but not integrated into training, which resulted in a 0.15% increase in new infection risk. These findings are in line with our initial expectations, where educated staff trained using an SOP reduced the new infection risk. Therefore, the result of this study supports our hypothesis that employee education and training have a positive impact on the new infection risk in dairy herds when those employees are involved in the day-to-day operation of the farm.

The rise in BTSCC when an SOP is available may initially seem counterintuitive because the application of a task-specific SOP describing the work procedure is an essential tool for providing training, but it needs to be integrated into the farm through training and be a herd-specific developed SOP [20]. Also, an SOP only indirectly affects the BTSCC because it is merely a description of the procedure, not a way to implement the procedure. Other researchers, Rodrigues et al. (2005) [36], have found that SOPs are essential for improving standardization when several different people are involved in the milking process, which, in the present study, ranged from one to twenty-one employees. But the first step in implementing an effective SOP at herd level is to develop it based on a structured framework, which includes, but is not limited to, involving the people there are going to use it in day-to-day operation, ensuring its available in languages necessary to reach all employees, doing a pilot test, and regularly evaluating if it reaches the target [21]. However, standardizing the procedures does not necessarily equate to a high-quality work standard. It is therefore essential when establishing an SOP to pay careful attention to the development process and approach to implementation before expecting any positive impact, for example, on the two dependent variables BTSCC and new infection risk [37]. Because there was a great diversity of nationalities on the enrolled dairy farms, the SOP provided were in Danish and or English, because a requirement for getting work permission is basic English skills. This requirement is of course very subjective, and they may only have basic language skills. The challenge with language is probably one of several reasons why the SOP is associated with higher BTSCC; others could be the method used for training or the culture of potential feedback on results.

The first step for the farmer hiring and introducing new personnel is to provide SOPs with the purpose of streamlining the work processes, but instigating and implementing the actual content of the SOP during training sessions for all employees is another challenge. The findings of De Treville et al. (2005) [38] showed that a lack of management and supervision reduced worker participation and lowered intrinsic motivation and creativity if workers did not participate in the development of the SOPs. Therefore, management should involve employees in herds with increased BTSCC in defining solutions to create awareness and engagement when working with udder health. The decrease in BTSCC was also positively associated with herd average ECM 305, in line with other researchers who identified an association between BTSCC and milk production [39]. We found more positive factors for new infection risk than for BTSCC, which can be expected because the impact from skills and procedures has more impact on controlling new infection risk, whereas BTSCC can more easily be controlled by management decisions such as actively separating milk or culling chronically infected cows not eligible for further treatment [12,40].

Other labor management studies included fewer participating farms than the current study [31]. These include a study focusing on labor management from Michigan [19] and a more recent study from Germany focusing on using an SOP as an integrated part of dairy farm management [22]. In the German study, the authors found a similar result in terms of significantly higher BTSCC on farms with an available SOP (mean ± SE: 201,168 ± 4579 cells/mL) than on farms without an SOP for standardizing the milking procedures (181,318 ± 7284 cells/mL; *p* = 0.023). This result is like the result in the current study, but the impact of the SOP was lower in the German study then in the present study. Our current study identified an association between udder health and employee education and management, where we have highlighted some aspects of human resource management that need to be improved to move forward in the dairy sector. Motivated individuals are committed to learning and have the potential to become long-term employees, and training is therefore essential for employee retention [33]. The dairy sector must embrace this fact and move away from relying on cheap, unskilled labor, which has been the norm in Europe for many years [41]. This is clearly illustrated in the present study, where educated employees are associated with decreased new infection risk. This is one of the challenges, as running a successful dairy operation cannot be accomplished only with employees on minimum wages, with limited attention to human resource management leading to an unacceptably high employee turnover and low quality of work performance. The turnover of employees must be addressed and quantified to uncover the reason, because we need to specify the reason. When an employee leaves, whether employer initiated or employee initiated, it may be beneficial if a less skilled worker leaves [42]. One way to implement a herd-specific SOP developed in cooperation with employees is by applying roadmaps as a teaching tool [43]. The herd veterinarian can play a role in this because the herd veterinarian is, after all, still the most critical consultant in working with udder health [44,45]. In the future, herd veterinarians must take a holistic 360-degree approach that includes all risk factors, including those of which we have limited experience and knowledge, to be able to improve udder health at the herd level. The NMC 10-point plan is a useful tool for this, but it is essential to incorporate the human aspects to improve and maintain udder health at the herd level.

## 5. Conclusions

We studied the association between udder health and the training of employees and employee management in 88 Danish dairy farms. Providing an SOP was found to have a positive association with BTSCC, increasing with 21,600 cells/mL, compared to herds with no SOP for milking, but the education level of the employees did not impact BTSCC. In addition, the herd average milk yield showed a negative association with BTSCC, with a decrease of 15,000 cells/mL for each 1000 kg increase in energy-corrected milk. We also identified a 0.02% reduction in new infection risk when the dairy farm used educated employees and a 0.16% decrease when an SOP was used in training process. The unskilled employees were associated with a 0.02% increase in new infection risk, training given by other employees with a 0.05% increase, training given by owners with a 0.11% increase, and an SOP available in the herd but not implemented by training with a 0.15% increase.

## Figures and Tables

**Table 1:** The questions and answer options included in the analyses.

Categories	Scale
Educational background	Skilled ^1^/Unskilled ^2^
Training sessions ^3^	Yes/No/Other ^6^
Training method ^4^	SOP/Training session/By example/Other
Work instruction (SOP) ^5^	Yes/No

^1^ Skilled—employee attending agricultural college. ^2^ Unskilled—employees from abroad/local area with no basic agricultural college training. ^3^ On-farm training with herd-specific material. ^4^ Training method—how the training is performed. ^5^ All milking tasks are explained in an SOP provided for all employees. ^6^ Cover that the respondent could not answer within the other defined categories.

**Table 2:** The explanatory and dependent variables of the regression and Poisson models.

Model	Explanatory Variable	Dependent Variable
1.	Herd variables ^1^	BTSCC
2.	Herd variables ^1^	New infection risk
3.	Management variables ^2^	BTSCC
4.	Management variables ^2^	New infection risk
5.	Management variables ^2^ + Herd variables ^1^	BTSCC
6.	Management variables ^2^ + Herd variables ^1^	New infection risk

^1^ Herd explanatory variables: herd average energy-corrected 305-day milk yield (Herd average ECM 305), herd average culling rate over 12 months (Culling rate), average herd size over the last 12 months (Herd size). ^2^ Management explanatory variables: educational background, training session, training method, work instruction.

**Table 3:** Descriptive statistics of 12-month geometric BTSCC and 12-month new infection risk.

	Mean	Median	Min.	Q1	Q2	Q3	Max.
BTSCC cells/mL ^1^	207,000	204,000	89,000	177,000	204,000	237,000	362,000
New infection risk ^2^	11	10	5	8	11	16	20

^1^ Herd average BTSCC 12 months before the date of the herd visit. ^2^ Herd average new infection risk in % over the 12 months prior to the date of the herd visit.

**Table 4:** The full and final model results with BTSCC as the dependent variable.

		Full Model			Final Model	
Coefficients	Estimates	95% CI	*p*-Value	Estimates	95% CI	*p*-Value
Intercept	179.872		0.00	196.614		
Skilled	1955	−11.128; 15.038	0.77			
Unskilled	5440	−604; 11.485	0.82			
Training other	−20.660	−111.109; 76.787	0.68			
Training SOP	13.108	−84.744; 110.959	0.79			
Training session	14.056	−26.371; 54.483	0.50			
No training	45.603	−74.043; 165.255	0.46			
Training other employees	11.516	−18.033; 41.066	0.45			
Training owner	5.480	−28.596; 39.955	0.75			
SOP provided	23.442	−1135; 48.019	0.07	21.590	507; 42.674	0.05
Herd size	−69	−143; 5	0.07			
Herd average ECM 305	−15.857	−25; −7	0.00	−15	−23; −7	0.00
Culling rate	12.380	−195.127; 219.886	0.90			

**Table 5:** The model results with new infection risk as the dependent variable.

		Full Model			Final Model	
Coefficients	Estimates	95% CI	*p*-Value	Estimates	95% CI	*p*-Value
Intercept	−1.67	−1.85; −1.49	0.00	−1.68	−1.85; −1.50	0.0
Skilled	−0.019	−0.03; −0.004	0.00	−0.02	−0.03; −0.00	0.01
Unskilled	0.019	0.012; 0.027	0.00	0.02	0.01; 0.03	0.00
Training, other	0.030	−0.144; 0.197	0.72			
Training, SOP	−0.161	−0.293; −0.033	0.02	−0.16	−0.29; −0.03	0.02
Training session	−0.001	−0.071; 0.049	0.74			
No training	0.019	−0.094; 0.032	0.30			
Training, other employees	0.005	0.013; 0.008	0.02	0.05	0.01; 0.08	0.01
Training, owner	0.011	0.006; 0.016	0.00	0.11	0.06; 0.16	0.00
SOP provided	0.015	0.012; 0.018	0.00	0.15	0.12; 0.18	0.00
Herd size	0	0; 0	0.03	0.00	0.00; 0.00	0.01
Herd average ECM 305	0	0; 0	0.00	0.00	0.00; 0.00	0.00
Culling rate	0.06	0.032; 0.09	0.00	0.60	0.32; 0.88	0.00

## Data Availability

The datasets presented in this article are not readily available because the data are part of an ongoing study, which will be published soon. Requests to access the datasets should be directed to the corresponding author.

## Supplementary Materials

The following supporting information can be downloaded at: https://www.mdpi.com/article/10.3390/vetsci11120646/s1, Figure S1: One the *x*-axis the number of educated employees in each dairy herd and on the *y*-axis the number of dairy herds. Figure S2: One the *x*-axis the number of uneducated employees in each dairy herd and on the *y*-axis the number of dairy herds. Illustrating how many uneducated employees there, and the number of herds enrolled. Figure S3: One the *x*-axis the training method used for training employees, and on the *y*-axis the number of dairy herds. Illustrating the method applied for trained in the dairy herd. Figure S4: One the *x*-axis the people responsible for training in the dairy herd, and on the *y*-axis the number of dairy herds. Illustrating by whom the employees are trained in the dairy herd. Figure S5: One the *x*-axis the distribution of dairy herds with and without a SOP, and on the *y*-axis the number of dairy herds. Illustrating the distribution of dairy herds without and with a SOP.
