# Supplementary material for: Employee Management in Dairy Farms Associated with Bulk Tank Somatic Cell Count and New Mastitis Infection Risk
Source: Vet Sci. 2024 Dec 13;11(12):646. doi: 10.3390/vetsci11120646 (PMC11680100; doi:10.3390/vetsci11120646)
Supplement: Supplementary file 1 [file vetsci-11-00646-s001.zip › vetsci-3309963-supplementary.pdf]

Supplementary Material

Article

Employee Management in Dairy Farms Associated with Bulk Tank Somatic Cell Count and New Mastitis Infection Risk

Michael Farre [1,*], Erik Rattenborg [1], Henk Hogeveen [2], Volker Krömker [3] and Carsten Thure Kirkeby [3]

[1] SEGES Innovation, Agro Food Park 15, 8200 Aarhus, Denmark; era@seges.dk
[2] Business Economics Group, Department of Social Sciences, Wageningen University and Research, 6706 KN Wageningen, The Netherlands; henk.hogeveen@wur.nl
[3] Department of Veterinary and Animal Sciences, Section for Production, Nutrition and Health, University of Copenhagen, 1870 Frederiksberg C, Denmark; volker.kroemker@sund.ku.dk (V.K.); ckir@sund.ku.dk (C.T.K.)
* Correspondence: mifa@seges.dk; Tel.: +45-23835400

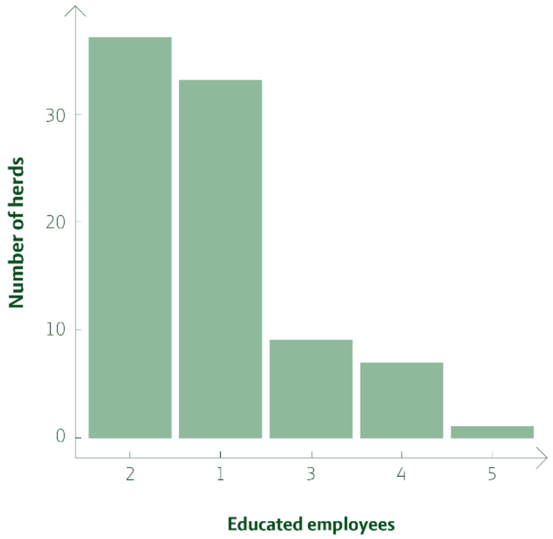

Figure S1. One the x-axis the number of educated employees in each dairy herd and on the y-axis the number of dairy herds. Illustrating how many educated employees there, and the number of herds enrolled.

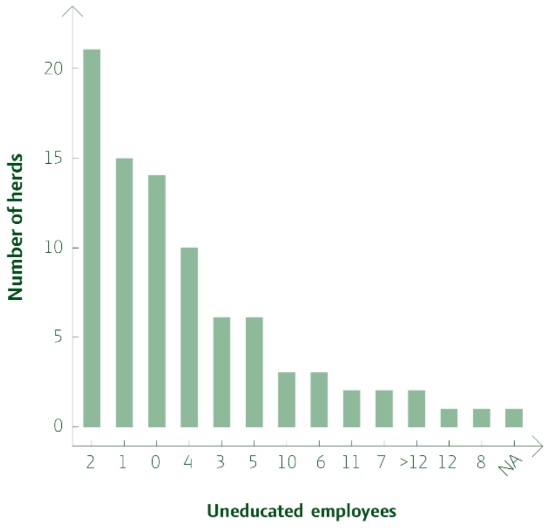

Figure S2. One the x-axis the number of uneducated employees in each dairy herd and on the y-axis the number of dairy herds. Illustrating how many uneducated employees there, and the number of herds enrolled.

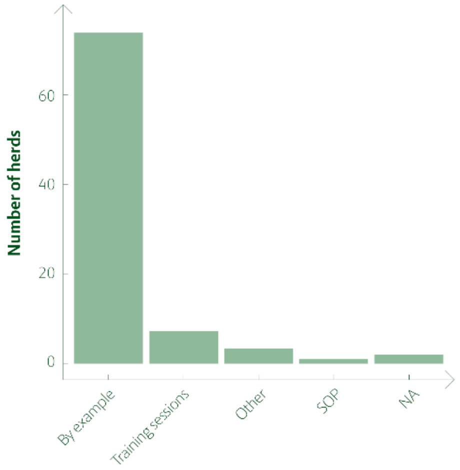

Figure S3. One the x-axis the training method used for training employees, and on the y-axis the number of dairy herds. Illustrating the method applied for trained in the dairy herd.

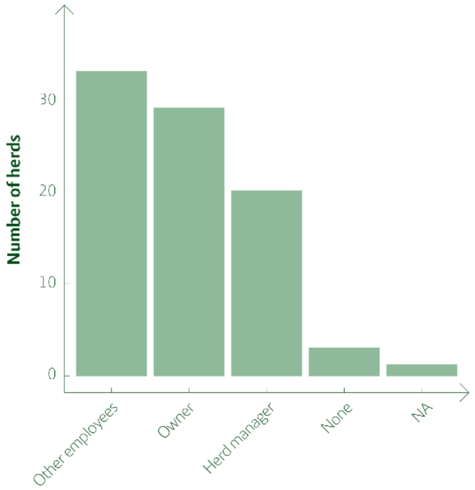

Figure S4. One the x-axis the people responsible for training in the dairy herd, and on the y-axis the number of dairy herds. Illustrating by whom the employees are trained in the dairy herd.

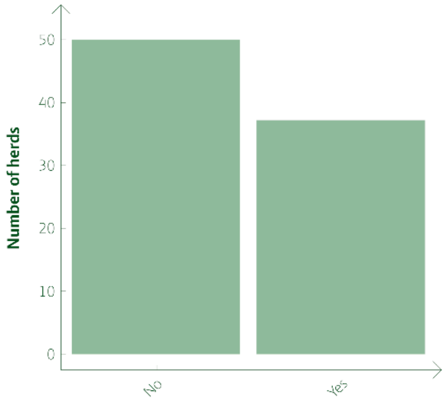

Figure S5. One the x-axis the distribution of dairy herds with and without a SOP, and on the y-axis the number of dairy herds. Illustrating the distribution of dairy herds without and with a SOP.
